# The Therapeutic Applications of Radiations

**Published:** 1914-03-21

**Authors:** 


### RADIUM AND RADIO-ACTIVITY.

### A SURVEY OF ITS THERAPY AND FINANCE.

### The Therapeutic Applications of Radiations.

There must always of necessity be topics which affect both the medical and the non-medical sections of the institutional world. At the present time this is pre-eminently true of treatment by radium. For the medical staffs of hospitals, and, indeed, for the whole medical profession, the exact powers and limitations of radium therapy are exceedingly important because they are so unlike anything which has hitherto been used in the perpetual struggle of man against his diseases. If the anticipations which have been formed concerning the possible future of radium therapy are fulfilled, or anywhere near fulfilled, much of modern medicine and surgery will have to be recast, and many recognised text-books will become obsolete. And apart from anticipations which are still more or less problematical, the actual performances of radium are of the greatest interest and importance. They are, as will be shown, by no means as successful yet as readers of the lay press might imagine; but they are in many ways remarkable.

To institutional managers radium therapy is chiefly of interest on account of the extremely high cost of radium and its compounds. The public has heard and read so much about radium that there is a tendency to despise as out of date or inefficient any institution which does not possess and use the drug. Boards of governors are naturally reluctant to incur the formidable expense entailed by the purchase of even a few milligrams of radium, unless it can be clearly shown that commensurate returns in the relief of human suffering are likely to accrue, and that they cannot otherwise be obtained. They turn to their medical staffs for information, and find conflicting opinions supplied to them. Meanwhile, under the pressure of public opinion, they are being steadily forced into bidding for the scanty supply of radium on the market, and so the price is driven higher and higher.

In these circumstances it has seemed that some good might be done, not only to the medical profession and the hospitals, but also to the interests of the lay public, by a collation of the opinions of those experts who are best entitled to express them. It is true that two eminent workers in radium therapy have quite recently expressed in public the view that it would be a good thing if, for the next two years, all their fellow-workers were bound or pledged not to publish results. With the spirit of this proposal, impracticable as it may be, it is impossible not to agree. It would be best for everybody if such a policy or conspiracy of silence could be secured. But so much has already been written about radium, both in the lay and the medical press, that a frank summary of the present position of knowledge in radium therapy may be of real service, especially when it is made plain how very small and uncertain that knowledge is, and how great is the resemblance between groping in the dark and treatment by radium at the present time. A further point of interest, both medically and institutionally, lies in the comparison of *x*-rays and radium. Both the *x*-ray tube and radium act upon human tissues by virtue of certain rays which they propagate. Those rays have certain points of similarity, as well as marked differences. They are used, broadly speaking, for very much the same classes of disease; and therefore comparisons of their efficacy are inevitable. Moreover, during the last ten years expensive *x*-ray installations have been provided at a great many hospitals up and down the United Kingdom, so that it is a point of vital interest to hospital treasurers and governors to know whether radium is likely to oust *x*-rays, or *x*-rays radium; or whether both are essential for the full and adequate equipment of a first-class hospital. So far the experts differ on this point; but the vast importance of arriving at an answer some day, and as soon as possible, needs no emphasising, for it is obvious.

#### Radium and its Emanation.

In the mid-'nineties Röntgen discovered that a Crookes' tube emits rays capable of passing right through all sorts of bodies (including the human frame) which are opaque to ordinary light, and able to act upon photographic plates and to manifest energy in other ways. Not long afterwards Becquerel discovered that rays having this same property of penetrating opaque substances are emitted from uranium compounds, and that this property is quite independent of any previous exposure to light. This power of spontaneously emitting rays is known as radio-activity, and such bodies as possess it are termed radio-active.

Schmidt and Madame Curie then discovered, independently of each other in 1898, that thorium compounds are radio-active, and eventually the latter demonstrated the existence of several new elements, of which radium is one, and by far the most remarkable. It was not, however, until four years ago that Madame Curie was able to isolate radium in a state of purity, though for some years before that a great deal of knowledge of the properties of this metal had been steadily accumulating. For fuller information upon this topic reference may be made to "The Radio-Active Substances," by Walter Makower, and to "Radium and Radio-Activity," by A. T. Cameron.

Compounds containing radium possess radio-activity in proportion to the percentage of the pure metal which they contain. Thus chloride of radium and carbonate of radium are more radio-active than bromide of radium, which is the salt most usually employed therapeutically, because they contain a higher ratio of the pure metal to the acid radicle. But besides emitting continuously certain rays, radium (and its compounds) emit also a gas, called "radium emanation," which is itself radio-active. This gas can be collected just as any other gas, and is itself now largely used in the treatment of disease. Insomuch that radium continues to give off this gas uninterruptedly for two thousand years before its own radio-activity is reduced to one-half, it is evident that the employment of the gas is a vastly cheaper matter than that of radium itself. If experience shows that the gas is equally efficacious, it is quite possible that in time the emanation may supplant the parent metal for therapeutic usage.

In some ways, as will be presently explained, radium emanation has actual advantages over solid radium and its salts. But it has one outstanding disadvantage, in that its radio-activity rapidly falls off, instead of remaining to all intents constant as in the case of radium itself. In 3.85 days from the time of its formation its power diminishes to one-half, and to half of that (*i.e.*, to one-quarter of the original) in a further 3.85 days, and so on. Therefore radium emanation must be used absolutely fresh, and in measuring the dosage allowance must be made for this rapid loss of power. That is, if radium emanation is used twelve hours old, let us say, the dose as made up for use must be rather stronger than that which it is desired actually to use. The rate of decay in strength being accurately known, it is a simple matter to calculate the extra dosage if it is known exactly how long after preparation the emanation is to be used.

The determination of the purity of a given sample of radium is a highly technical and difficult matter. Yet there is so much "radium" on the market which—if not purposely adulterated—is very far from pure, that it is essential for such tests to be undertaken before any specimen is paid for. This is a point to which institutional authorities should pay most particular attention. No consignment should be accepted in future which does not contain at least 90 per cent. of a pure salt of radium. This minimum is one which there is no difficulty in attaining, though it is probably true that few institutions in this country have in fact secured radium of such a high degree of purity. With regard to radium emanation there is not the same danger of employing strengths less than is realised, because any institution (or private practitioner) possessing a stock of radium will find it advantageous and comparatively simple to manufacture home-made emanation. The unit of emanation is known as a *curie*, in honour of the distinguished French lady scientist; and a solution of radium emanation for medical use should have a strength of at least one *millicurie* per litre.

